# What is the abdomen? Rationalising clinical and anatomical perspectives using formal semantics

**DOI:** 10.1111/joa.13384

**Published:** 2021-01-08

**Authors:** Philip J. B. Brown, Yongsheng Gao, David Clunie

**Affiliations:** ^1^ Classicum Norwich UK; ^2^ SNOMED International London UK; ^3^ PixelMed Publishing LLC Bangor PA USA

**Keywords:** abdomen, abdominal cavity, anatomy, biological ontologies, pelvis, radiology metadata, systematized nomenclature of medicine, thorax

## Abstract

The meaning of the term ‘abdomen’ has become increasingly ambiguous, as it has to satisfy the contemporary requirements of natural language discourse, literature, gross and radiological anatomy and its role in ontologies supporting electronic records and data modelling. It is critical that there is an agreed understanding of the semantics of the abdominopelvic cavity, its component volumes including the abdomen proper, true and false pelvic cavities, and its boundaries and regional contents. The expression of part–whole (meronymic) relationships is essential for inferences to be drawn by computer algorithms, but unless these are rigorously reviewed and tested incorrect assumptions are drawn. The SNOMED CT terminology descriptions and hierarchy of anatomical concepts relating to the trunk were scrutinised for ambiguity and sub‐optimal relationships using a panel of reference sources. Any identified errors were corrected and the impact of any changes reviewed iteratively by evaluating their effect on dependant hierarchies (modelled with the associated anatomical concepts). Anatomical concepts are generally structured according to a traditional gross standpoint, but in clinical practice covert complex regional notions are frequently used and during the evaluation process a new viewpoint relating to projectional (transmissive) or emissive radiological perspective was identified. The subtle but important differences in the boundaries, volumes and contents of these distinctive perspectives of the ‘abdomen’ are presented. Three significant complex variants have been identified which relate to the most common uses of the word ‘abdomen’. The merits and disadvantages of using ‘abdomen’ as common synonym to more than one concept (polysemy) are briefly discussed and the solution adopted by SNOMED International described. The review of existing ontologies and academic literature confirmed the frequent varied use of the word ‘abdomen’, which raises concerns when derived data are increasingly being used remotely from the point of clinical contact, potentially leading to incorrect inferences. The documented regional truncal volumes from an anatomical regional, segmental and cross‐sectional perspective have been integrated into a logical and comprehensive model suitable for computer processing. The robust modelling of meronymic hierarchies has to be rigorous to avoid systematic errors and it is thus timely that a proposed standard description of these subtly related volumes and structures is made available for discussion and comment.

## Graphical abstract

The meaning of ‘abdomen’ is ambiguous in modern discourse and literature. Ontologies are being used to capture, model and process data: a lack of clarity of the meaning of ‘abdomen’ can cause incorrect inferences when derived data are used remotely from the point of clinical contact. A set of defined regional truncal concepts from an anatomical, segmental and cross‐sectional perspective have been integrated into a logical and comprehensive model, suitable for computer processing.

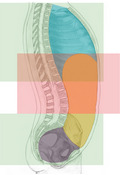

## 1 INTRODUCTION

Historically the clinical notion of the abdomen was coterminous with its gross anatomy and Gray (1918:1147) asserts that the cavity's ‘upper extremity is formed by the diaphragm’ and its lower extremity is ‘formed by the structures which clothe the inner surface of the bony pelvis, principally…the diaphragm of the pelvis’; Gray goes on to state that ‘In order to facilitate description, it is artificially divided into two parts: an upper and larger part, the abdomen proper; and a lower and smaller part, the pelvis…These two cavities are not separated from each other, but the limit between them is marked by the superior aperture of the lesser pelvis’.

The ambiguous use of the term ‘abdomen’, to mean abdominopelvic or abdomen proper structures has continued and become more prevalent in both clinical practice and the literature. For example, the term ‘intra‐abdominal abscess’ is most commonly used, rather than ‘intra‐abdominopelvic abscess’, to describe the collection of pus or infected material within the abdominal and/or pelvic cavities (https://bestpractice.bmj.com/topics/en‐gb/996; Jan van Oss, 2009:336; Mehta & Copelin, 2019; Park & Charles, 2012:311; Schein, 2001). The practice of using the term ‘abdomen proper’ for clarity is also now uncommon, having increasingly fallen out of general use. Thus, ‘abdomen’ in natural language is used to refer to a number of concepts, including but not limited to, the following homonyms:

- Abdominopelvic cavity.
- Abdominopelvic cavity excluding the true pelvic cavity (abdomen proper cavity).
- Abdominopelvic cavity and/or content (intra‐abdominopelvic structure).
- Intra‐abdominopelvic structure excluding intra‐pelvic structure of true pelvis.
- Intra‐abdominopelvic structure and/or anterior abdominal wall.
- Intra‐abdominopelvic structure and/or anterior abdominal wall, excluding intra‐pelvic structure of true pelvis (abdomen proper).
- Abdominal segment of trunk.

The meaning of the word ‘abdomen’ may be more clear during dialogue between individuals knowledgeable of the context of its use, but ambiguity is increasingly prevalent the more remote the discourse, such as in shared clinical records.

A number of existing schema support the coding of anatomic entities:

- Terminologia Anatomica ‐ Second Edition, Federative International Programme for Anatomical Terminology of the International Federation of Associations of Anatomists (https://fipat.library.dal.ca/ta2/).
- Foundational Model of Anatomy (FMA) v.5; http://fma.si.washington.edu/browser/#/).
- Uberon (http://uberon.github.io).
- International Classification of Diseases 11th Revision (ICD‐11) anatomy and topography extension codes, 2019 (https://icd.who.int/browse11/l‐m/en).
- Radiology Lexicon (RadLex) (http://radlex.org).
- Medical Subject Headings (MeSH), 2019 (https://meshb.nlm.nih.gov/search).

Terminological Anatomica (TA) (FIPAT, 2019) provides an authoritative standardised nomenclature for the naming of anatomical structures but lacks a detailed hierarchical structure. Uberon by contrast is an anatomical ontology for a variety of animal species, with a focus on vertebrates (Haendel et al., 2009; Mungall et al., 2012) which is rich in partonomic relationships but is incomplete in human anatomical regions, for example, the pancreas (https://www.ebi.ac.uk/ols/ontologies/uberon/terms?iri=http%3A%2F%2Fpurl.obolibrary.org%2Fobo%2FUBERON_0001264) is placed as a viscus of the trunk but not within the abdomen. FMA provides more clarity with a detailed ontology of anatomical entities and their subclass and part‐of relationships that has also been used as a source for other terminologies such as RadLex, which uses a derived subset of anatomical concepts relevant to the domain of radiology (Mejino et al., 2008). A review of these schemas (Table 1) reveals that there is no existing uniformity of the meaning of ‘abdomen’: TA regards this term to relate to the abdomen excluding the pelvis (abdomen proper), as does MeSH; in contrast, FMA (and by derivation RadLex) considers the abdomen to be inclusive of the abdomen and pelvis; Uberon utilises abdominopelvic region as a synonym but has a conflicting definition that excludes the pelvis; the ICD‐11 extension code specifies the abdomen as a lower trunk ‘surface topography’ but does not stipulate the relationship of the pelvis with the trunk, resulting in uncertainty.

**TABLE 1:** The notion of ‘abdomen’ as represented and defined by different coding schema

Schema (code)	Term [synonym]	Definition
TA (127)	Abdomen	*No definition: but specifies pelvis as a separate sibling entity thus indicating that this notion relates to the abdomen proper*.
Uberon (0000916)	Abdomen [abdominopelvic region]	The subdivision of the vertebrate body between the thorax and pelvis. The ventral part of the abdomen contains the abdominal cavity and visceral organs. The dorsal part includes the abdominal section of the vertebral column.
FMA (9577)	Abdomen [abdominopelvis, abdominopelvic region]	Subdivision of front of trunk, each instance of which has as its constitutional part some complete set of lumbar vertebral arches (L1‐L5); it is demarcated from the abdomen internally by the superior surface of the diaphragm and externally by the costal margin and from the pelvis by the plane of the superior pelvic aperture; together with the abdomen and pelvis constitutes the trunk.
RadLex (RID56)	Abdomen [abdominopelvis]	Subdivision of trunk proper which is demarcated from the thorax internally by the inferior surface of the sternocostal part of the diaphragm and externally by the costal margin, from the back of abdomen by the external surface of the posterior abdominal wall, from the perineum by the superior surface of the urogenital diaphragm and from the lower limbs by the inguinal folds; together with the thorax, and perineum, it constitutes the trunk proper.
ICD‐11 (1983193090)	Abdomen [Abdomen NOS]	*No definition: pelvis is not a subordinate of abdomen or lower trunk, thus, it is unclear if this means abdominopelvic region or abdomen proper*.
MeSH (D000005)	Abdomen	That portion of the body that lies between the thorax and the pelvis.

FMA is an important standard against which SNOMED Clinical Terms (SCT) is measured and shares considerable content: FMA is described as a domain ontology that represents a coherent body of explicit declarative knowledge about human anatomy (http://si.washington.edu/projects/fma); in contrast, SNOMED CT is specifically designed to directly support healthcare implementations and uses anatomy to underpin the clinician's view of the world, which is frequently from a regional perspective. The most common clinical use of ‘abdomen’ when referring to disorders and procedures relates to anatomical structures within the abdominopelvic cavity and the anterior abdominal wall. FMA models the abdomen (http://purl.org/sig/ont/fma/fma9577) from a pure anatomical standpoint and considers it to have regional components of the ‘abdomen proper’ and the ‘pelvis’; and constitutional parts including the lumbar vertebral column as part of the posterior abdominal wall: while this may be technically correct clinicians would not consider disorders and procedures of the lumbar vertebrae to fall within the area of the ‘abdomen’. In addition, although FMA has a rich partonomy, there are some important anatomical structures that clinicians regard as being within the ‘abdominal region’ that are excluded, for example, inguinal canal, skin of umbilicus.

Currently, while these coding schemas’ handling of the notion of abdomen may be sufficient for the purposes of their domain requirements, there remains an imperative requirement for semantic‐based concepts, which unambiguously describe these clinical regional anatomical notions, context free, for use within ontologies, clinical records and documents.

SNOMED CT is a dynamic clinical terminology and is updated and released by SNOMED International every 6 months, to respond to healthcare advances and user feedback. These changes require the terminology to undergo continuous review, augmentation and modification. The content is modelled using formal Web Ontology Language (OWL) 2 syntax (SNOMED CT OWL Guide, 2020b, available at http://snomed.org/owl; Grau et al, 2008; http://www.w3.org/TR/owl2‐syntax/) to represent logical semantics. The release format includes tab delimited text files that are commonly used by EHR implementations; in addition, the stated logical axioms are also released in OWL syntax conforming to the OWL 2 EL profile (SNOMED CT Logic Profile Specification, 2020a, available at http://snomed.org/lps).

As part of this continual review and quality assurance activity, it was recognised that some clinical procedure and disorder relationships were suboptimal with respect to distinguishing the anatomical subtleties of different homonyms of the term ‘abdomen’, with some discrepancies between the clinical usage of terms and their apparent semantics in specific clinical domains.

This study describes the process followed to evaluate the existing SNOMED CT anatomical concepts and hierarchies related to the main regions of the trunk, viz. thorax, abdomen and pelvis, with reference to existing ontologies and published anatomical benchmark literature.

The objectives of this enquiry were to:

- evaluate the existing content and relationships of the anatomical concepts within SNOMED CT relating to the trunk region, by reviewing all terms, semantic definitions and locations in the class hierarchy;
- identify relevant regional classes of anatomical concepts used within clinical discourse, for example, new constructs to support cross‐sectional, projectional and emissive radiological procedures;
- develop a set of logically defined concepts, representing the most important clinically relevant regional volumes, relating to the abdominopelvic region that support healthcare (including radiological) perspectives;
- integrate the revised set of regional anatomical concepts within SNOMED CT and evaluate their relationships within their semantic neighbourhood and
- re‐evaluate and quality assure the relevance of these regional concepts by scrutinising their impact on dependant disorder and procedure hierarchies by identifying the subsumption of concepts that were previously missed and inadvertently included.

### 1.1 Terminology standards

Standards for anatomical terminology has a long history and the International Federation of Associations of Anatomists produced the first Basle Nomina Anatomica (BNA) in 1895 (His), which had 5528 terms: this underwent a series of editions, and expansion to contain 5640 terms, to become the Nomina Anatomica in 1955 (Woerdeman, 1957); and subsequently developed into the Terminologia Anatomica in 1998 (FCAT) with 9200 terms within its first edition. The Terminological Anatomica (TA) is now in its Second edition (2.02) and distinguishes the abdominopelvic cavity (3699) from the abdominal cavity (3700) and pelvic cavity (3701); the word abdomen (127) excludes the pelvis and thus relates to the ‘abdomen proper’, although this phrase is not overtly used.

With the advent of computers and the electronic health record in the 1980’s, it was recognised that it was not only imperative to have a standard anatomical vocabulary but that the identified concepts required both an agreed preferred term and explicit relationships to each other, defined by formal semantics. Terminologies were developed for this purpose including SNOMED (Cote & Rothwell, 1989); the Read codes (Chisholm, 1990); Clinical Terms Version 3 (Schulz et al., 1997); GALEN (Rector et al., 2000); SNOMED RT (Spackman et al., 1997) and later, building on these earlier initiatives, the Foundational Model of Anatomy ontology (FMA; Rosse & Mejino, 2008).

The anatomy hierarchy in Clinical Terms Version 3 (CTV3) was developed by a panel of clinicians and anatomists in harmony with the Nomina Anatomica (Schulz et al., 1997), and distinguished the ‘abdominal cavity proper structure’ as a separate concept from the ‘pelvic cavity structure’; the FMA subsequently similarly identified ‘abdomen proper’ as a discrete concept (http://purl.org/sig/ont/fma/fma61680) and defined it as a ‘subdivision of abdomen which is demarcated from the pelvis by the plane of the superior pelvic aperture’.

In 2000, CTV3 and SNOMED RT merged (Wang et al., 2001) to create SNOMED Clinical Terms (SNOMED CT) in which concepts are identified by a unique numerical concept identifier; a nominally unambiguous ‘fully specified name’ (FSN); and one or more synonymous terms (one of which is specified as the preferred clinical description). In SNOMED CT, text definitions are rarely assigned to concepts, but most clinical concepts are modelled semantically using detailed sets of ‘values’ including anatomy, organisms and morphologies, etc. The logical structuring of these values (especially anatomy), according to their meaning into multiple hierarchies, is important as they form the basis of automatically generating the relationships between clinical concepts such as disorders and procedures (using auto‐classification) and are critical for data input and subsequent information retrieval and for decision support (Brown & Sönksen, 2000).

## 2 METHODS

The anatomical concepts of SNOMED CT are arranged according to published rules (SNOMED International Editorial Guidelines, 2020, available at http://snomed.org/eg), which are in keeping with other earlier schemas including Clinical Terms Version 3 (Schulz et al., 1997) and GALEN (Rector et al., 2000). Two key models are particularly pertinent.

### 2.1 Body – Wall – Cavity – Contents model

In basic terms, the human trunk is formed from a series of volumes bounded by walls and enclosing structural content; for example, the thoracic cavity is bounded by the thoracic wall and contains structures including the heart and lung. The boundaries of an anatomical volume can be virtual, for example, superior thoracic aperture, or structural, for example, thoracic diaphragm. The FMA defines the body wall as a ‘subdivision of trunk that consists of those organs that separate the body cavity from the body's exterior; together with the body cavity and its contents, the body wall constitutes the trunk.‘ The FMA also considers the wall as a constitutional part of the ‘compartment’ (along with the space and content): The approach in SNOMED CT historically has been subtly different by using the word ‘compartment’ to describe the space and content only (but not the wall). In order to avoid confusion and provide clarity, the term ‘intra’ has been adopted to describe the combination of space and contents, as illustrated in the following amended SNOMED CT anatomy is‐a hierarchy (where x means any anatomy):

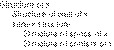

For example, in SNOMED CT, ‘intra‐abdominopelvic structure’ subsumes the ‘structure of abdominopelvic cavity’ and ‘structure of organ within abdominopelvic cavity’ and other contents.

### 2.2 Meronymic reasoning: Structure – Entire – Part (SEP) model

Taxonomic knowledge is a major portion of medical ontologies, and is mainly characterised by generalization and part–whole (meronymic) relations between concepts (Schulz et al., 1998). Generalised relationships are classically managed by expressing is‐a associations between two concepts and any information instantiated at a superordinate level is true for all its subordinates (Smith & Medin, 1981). Part–whole reasoning is more complex and SNOMED CT deals with this by using a SEP (Structure, Entire, Part) model where the anatomy hierarchy differentiates classes of entire anatomical entities from classes of parts of entire anatomical entities, for example:

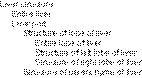

The above hierarchy of structure concepts is the consequence of logical classification by the DL reasoner and the following is a list of simplified logical definitions in OWL 2 Manchester Syntax:

- Class ‘Liver structure’ EquivalentTo ‘All or part of’ some ‘Entire liver’
- Class ‘Entire liver’ SubClassOf ‘Entire organ’ and ‘Constitutional part of’ some ‘Entire abdomen proper’ and ‘Systemic part of’ some ‘Entire digestive system’
- Class ‘Liver part’ EquivalentTo ‘Proper part of’ some ‘Entire liver’
- Class ‘Structure of lobe of liver’ EquivalentTo ‘All or part of’ some ‘Entire lobe of liver’
- Class ‘Entire lobe of liver’ SubClassOf ‘Regional part of’ some ‘Entire liver’
- Class ‘Structure of parenchyma of liver’ SubClassOf ‘Constitutional part of’ some ‘Entire liver’

An ‘entire concept’ denotes a class that is instantiated by entire anatomical entities of some kind, for example, entire liver is instantiated by all individual livers.

A ‘part concept’ denotes a class that is instantiated by all anatomical entities that are a proper part of some entity of a given kind, for example, a ‘liver part’ is instantiated by all entities that are proper parts of some liver, for example, my left lobe of liver, your middle right Hjortso liver segment; but a ‘liver part’ is not instantiated by any liver.

The ‘structure of x’ concept denotes a class that is the aggregation of an entire entity and/or its parts, which is commonly used in clinical practice and it provides convenience to assist with the logic‐based definitions and queries; therefore, ‘liver structure’ represents the entire liver and/or any part of the liver.

In SNOMED CT, the structure concept can also represent either a cavity or its contents enabling the support of modelling any condition or procedure that involves the cavity, content or both. In this way, the notion of ‘structure’ includes ‘immaterial’ cavity and ‘material’ content, even though they are disjoint anatomical entities.

An essential principal is that a structure cannot be a sub‐concept of another structure unless the anatomical entity lies entirely within the boundary of the superordinate structure. For example, the aorta cannot be subordinate to the thoracic structure as its abdominal segment lies outside; by contrast, the arch of the aorta lies entirely within the boundaries of the thorax and consequently is a constitutional part of that structure. Using this logic, the liver structure is part of the ‘abdominal proper structure’ and as this latter concept is subsumed by the abdominopelvic structure, it can be instantiated that the liver is also an abdominopelvic structure, as illustrated below:

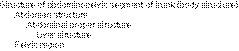

A disorder of the abdominopelvic segment structure represents a class of disorders in the abdomen, pelvis or both of them. Therefore (derived from the structure hierarchy above), a ‘disorder of liver’ is a disorder of the abdomen proper and also a disorder of abdominopelvic segment of trunk:

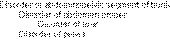

### 2.3 Auto‐classification

SNOMED CT concepts are semantically defined with description logic (DL) utilising comprehensive value hierarchies, notably anatomy. These definitions are applied by SNOMED CT authors within an Authoring Platform producing a ‘stated form’ in OWL 2 syntax that defines each concept and is also identified as being either completely defined when all the necessary characteristics are sufficiently expressed, or ‘primitive’ when the definition is incomplete. These definitions are then utilised by a software ‘DL classifier’ to organise the concepts logically into hierarchies. All and only concepts satisfying the definition of a higher‐level ‘ancestor’ concept are classified under it as ‘descendants,’ and everything said within SNOMED CT about any concept applies to all of its descendants.

This auto‐classification mechanism means that when a change is made to the anatomy hierarchy, its impact can be visualised by examining whether the resultant effect on dependent hierarchies such as procedures and disorders is logical.

### 2.4 Evaluation of existing concepts and hierarchies

The anatomical value hierarchy of SNOMED CT (January 2019 release version) below the concept ‘Trunk structure’ (http://snomed.info/id/22943007) was manually reviewed to evaluate each concept's existing FSN, preferred term, synonyms, semantic definition and hierarchical location. The majority of the content was uncontentious but there were a number of concepts that used the term in the form ‘* of abdomen’ or ‘Abdominal *’ (where * is a wild card of a range of characters): These concepts related more frequently to notions concerning both the abdomen and pelvis (abdominopelvic region) as apposed to the ‘abdomen proper’ (abdominopelvic region excluding the true pelvic cavity). Some were found to be ambiguous (e.g. structure of abdominal organ, abdominal cavity structure, abdominal structure, structure of fascia of abdomen), in being unclear as to whether they were referring to the abdominopelvic region, or exclusively to the abdomen proper, that is, excluding the pelvic cavity and contents.

The semantics of these concepts were compared to the following existing resources: TA, FMA, Uberon, RadLex, MeSH and ICD‐11. Anatomical textbooks, for example, Gray's anatomy (Standring, 2015) were also referenced, and scientific publications were consulted to gain insight into the use of a term. When there was no identified agreement of the meaning of a term, for example, pelvic diaphragm or posterior abdominal wall, specialist opinions were obtained to achieve consensus. The outcome of this exercise was the identification of a small subset of regional concepts whose meaning required improved clarity in expression.

Great effort and emphasis are placed in SNOMED CT in creating an FSN that fully and unambiguously conveys the meaning of the concept; but despite this aspiration enhanced precision is required when defining some clinical regional volumes, especially their borders and which of these boundaries are included within the concept. For example, even though the thoracic diaphragm and the pelvic diaphragm form the respective superior and inferior boundaries of the abdominopelvic cavity, only the former is generally considered a part of the structure.

### 2.5 Development and integration of new regional concepts

During this initial evaluation, a number of additional regional concepts were identified relating to truncal segments, the abdominopelvic cavity and its divisible volumes of the abdomen proper and true pelvic cavities, which required expression with FSNs and textual definitions. These anatomical concepts were added into the existing SNOMED CT hierarchy and appropriate regional and ‘part of’ subordinates placed beneath. This new organisation was then used to re‐classify the existing ‘finding’ and ‘procedure’ concepts, defined using these values, to assess the impact on dependant relationships. Further editing of the value hierarchies was performed in response to address any inconsistencies identified. This process was iterative, repeatedly reviewing the impact of increasingly minor changes and revising the anatomy accordingly.

Following the release of these initial amendments, an external expert (DC) alerted the SNOMED CT authoring team that the revised anatomy did not adequately support some radiological procedures such as Magnetic Resonance Imaging (MRI) and Computed Tomography (CT). Investigation and discussions highlighted that in diagnostic radiology the term ‘abdomen’ can be used quite specifically in the context of imaging procedures. Such procedures are cross‐sectional and the radiological convention has usually been to use the abdomen to mean the full thickness of the abdomen, excluding the pelvis, which was inconsistent with the proposed updated SNOMED CT model. The outcome of these discussions was the recognition of a new class of regional anatomical cross‐sectional volumes of the thorax, abdomen and pelvis and their constituent parts. This new set of cross‐sectional concepts have been defined and integrated within the existing SNOMED CT hierarchy, and then evaluated by again scrutinising their impact on dependant disorder and procedure classification. Analyses of the impact results identified false‐negative and false‐positive concepts due to: suboptimal definition of the dependant concepts; missing subordinate anatomy or inadvertent inclusion of concepts within new regional volumes. Concepts identified with suboptimal placement were corrected through a series of iterative audit impact cycles to validate and quality assure the finalised integrated regional anatomical hierarchy.

This process identified that the most common interpretations of ‘abdomen’ in clinical practice frequently related to quite complex regional anatomical volumes: These key volumes are described in gross anatomical terms and have been allocated standardised FSNs, preferred terms, definitions and hierarchical locations in SNOMED CT with accompanying illustrations in the next section.

## 3 RESULTS AND DISCUSSION

This section defines the regional and constitutional parts of the ‘trunk structure’ and the anatomical structures contained within the different specified volumes. The descriptions delineate the stated volume's clinically relevant boundaries, walls, cavities, segments and structures by specifying a unique and unambiguous ‘fully specified name’ (FSN), a textual definition along with their relationships with other related concepts.

### 3.1 Anatomical boundaries and planes of the trunk

**FIGURE 1:**
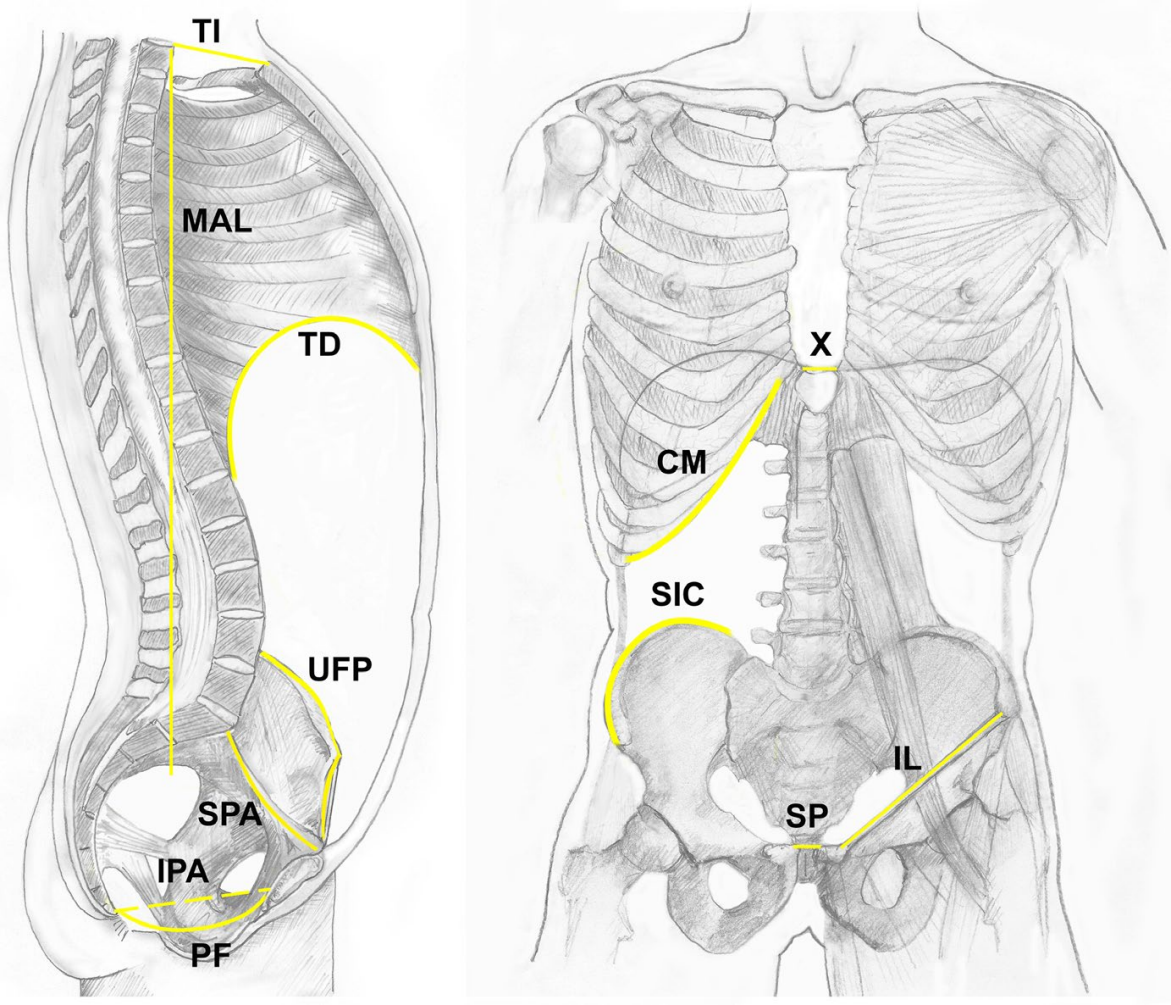
Boundaries of the trunk. The main boundaries of the trunk are indicated: CM, costal margins; IL, inguinal ligament; IPA, inferior aperture of true pelvis; MAL, mid‐axillary line; PF, pelvic floor; SIC, superior iliac crest; SP, symphysis pubis; SPA, superior pelvic aperture; TD, thoracic diaphragm; TI, thoracic inlet; UFP, upper border of false pelvis; X, xiphisternal joint

Figure 1 illustrates the main relevant anatomical morphological boundaries and planes of the trunk including the:

- Thoracic inlet – a virtual boundary (also known as the superior thoracic aperture) delimited by the first thoracic vertebra (T1); the first pair of ribs laterally and the costal cartilage of the first ribs which are continuous with the superior border of the manubrium and the chest wall anteriorly;
- Thoracic diaphragm – (usually abbreviated to the ‘diaphragm’);
- Upper border of false pelvis – a virtual plane from the symphysis pubis to the superior iliac crests; it is incomplete anteriorly, presenting a wide interval between the anterior borders of the pelvic ilia;
- Superior pelvic aperture – a virtual curved oblique plane passing through the sacral promontory posteriorly and the lineae terminales laterally, composed of the iliac arcuate line, pectineal line (pecten pubis), to the pubic crest. It is also known as the pelvic inlet or pelvic brim. Anatomically, the sacral promontory is sometimes situated above the posterior projection of this plane. This plane separates the abdomen proper cavity from the true or minor pelvic cavity;
- Inferior aperture of true pelvis – a trapezoidal shaped inferior aperture of the true (minor) pelvis and its boundaries are formed: posteriorly by the coccyx; laterally by the inferior pubic ramus, the ramus of the ischium, the ischial tuberosity and the sacrotuberous ligament; and anteriorly by the symphysis pubis. A transverse virtual (inter‐ischial) line between the ischial tuberosities divides the region into a posterior anal triangle related to the anus and an anterior urogenital triangle, which relates to the external urogenital organs and
- Pelvic diaphragm – a structure spanning the inferior opening (Key, 2010) with the musculotendinous diaphragm attached to the inferior aperture separating the cavity of the true pelvis from the perineum.

### 3.2 Cavities of the trunk

**FIGURE 2:**
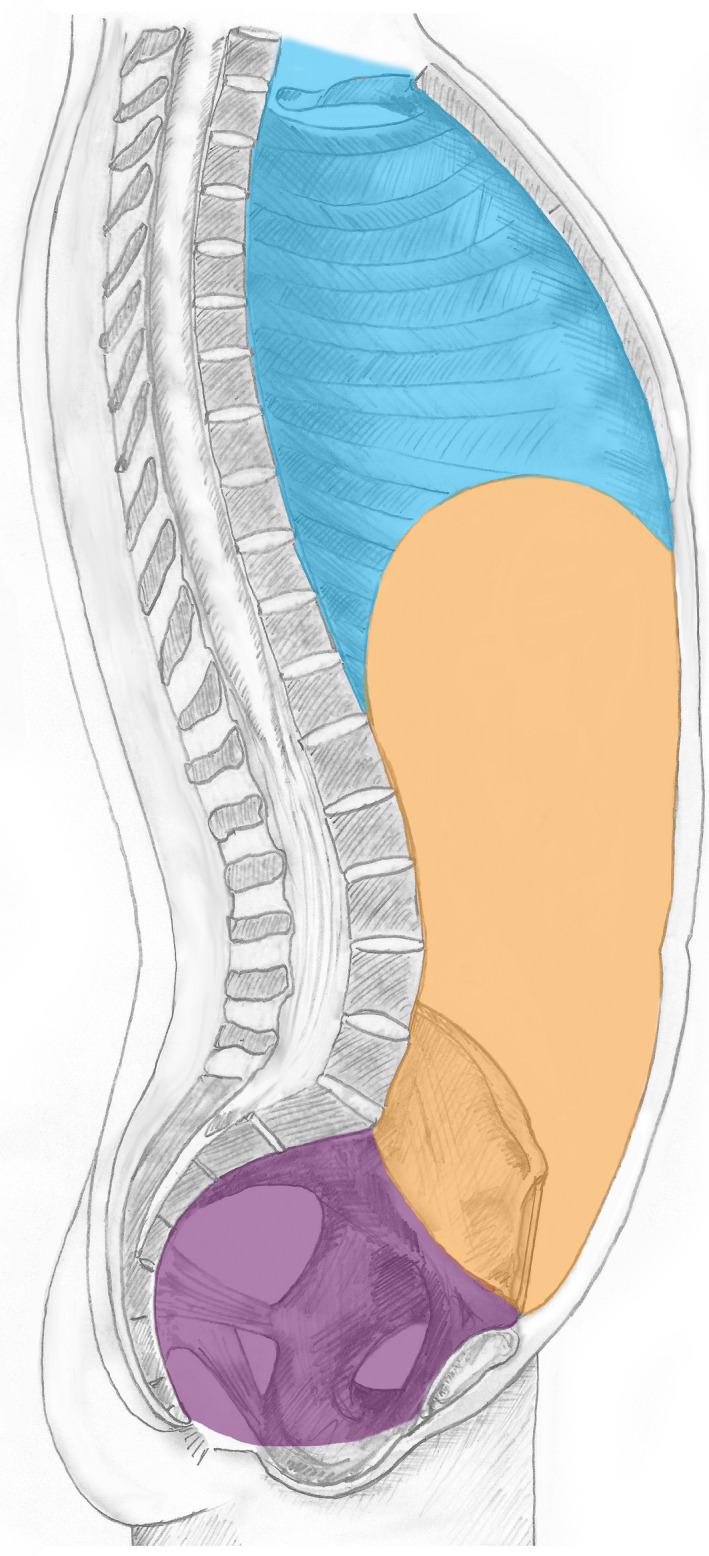
Cavities of the trunk. The illustration identifies the thoracic cavity http://snomed.info/id/43799004 (cyan), abdomen proper cavity http://snomed.info/id/281902004 (amber), cavity of true pelvis http://snomed.info/id/816991004 (purple) and the abdominopelvic cavity http://snomed.info/id/818987002 (amber and purple). The hyperlinks detail the numerical concept identification (conceptid) relating to the applicable concept in SNOMED CT

The planes described above form significant boundaries of the following anatomical cavities of the trunk, specified by their SNOMED CT preferred tem and FSN in parentheses (Figure 2):

#### 3.2.1 Thoracic cavity (Thoracic cavity structure)

This cavity is bounded superiorly by the thoracic inlet (superior thoracic aperture); inferiorly by, but excluding, the thoracic diaphragm; and laterally by, but excluding, the thoracic wall (highlighted with cyan in Figure 2).

#### 3.2.2 Abdominopelvic cavity (Structure of abdominopelvic cavity)

This cavity is bounded by, but excludes: superiorly the thoracic diaphragm; inferiorly the pelvic diaphragm; anteriorly the anterior abdominal wall and posteriorly the ‘posterior wall of abdomen proper’ (highlighted with amber and purple in Figure 2).

The abdominopelvic cavity subsumes smaller sub‐volumes that extend between the ‘abdomen proper’ and true pelvic cavities, including the:

- Peritoneal space – which lies between the parietal peritoneum (that lines the abdominal wall) and the visceral peritoneum (that surrounds the internal abdominopelvic organs) and
- Extraperitoneal space – which lies outside the peritoneum and can be divided into the:
  - Retroperitoneal space, situated posteriorly to the peritoneum;
  - Preperitoneal space, situated anteriorly to the peritoneum (including the retropubic and retroinguinal space);
  - Subphrenic extraperitoneal space;
  - Prevesical and perivescical space and
  - Perirectal space

#### 3.2.3 Abdomen proper cavity (Structure of abdominopelvic cavity excluding true pelvic cavity)

This cavity is bounded by, but excludes: superiorly the thoracic diaphragm; inferiorly the superior pelvic aperture; anteriorly the anterior abdominal wall and posteriorly the posterior wall of abdomen proper (highlighted with amber in Figure 2).

#### 3.2.4 Cavity of true pelvis (Structure of cavity of true pelvis)

This bowl‐shaped volume, also known as the cavity of the minor or lesser pelvis, is bounded by, but excludes: superiorly the superior pelvic aperture; inferiorly the pelvic diaphragm and laterally the pelvic wall (highlighted with purple in Figure 2).

#### 3.2.5 Cavity of false pelvis (Structure of cavity of false pelvis)

The cavity of the false pelvis, also known as the cavity of the major or greater pelvis, is bounded by, but excludes: superiorly an artificial plane named the ‘upper border of false pelvis’ (that extends from the symphysis pubis to the superior iliac crests); and inferiorly the superior pelvic aperture. It is bounded on either side by the ilium, which is the expanded portion of the bony pelvis above and in front of the superior pelvic aperture (Chung & Chung, 2008): in front it is incomplete, presenting a wide interval between the anterior borders of the ilia. It forms the inferior volume of the abdomen proper cavity.

#### 3.2.6 Cavity of false and/or true pelvis (Structure of cavity of false and/or true pelvis)

This more general notion of the pelvic cavity is sometimes used, for example, in obstetrics, which combines the two volumes described above, that is, the cavity of true pelvis (lesser pelvic or minor pelvic cavity) and the cavity of false pelvis (greater pelvic or major pelvic cavity). This cavity is bounded by, but excludes: superiorly an artificial plane the ‘upper border of false pelvis’; inferiorly the pelvic diaphragm and laterally the pelvic wall. The bony pelvis extends superiorly with the ilia, which anteriorly are incomplete, presenting a wide interval between the anterior borders of the ilia. The volume can be divided into the superior false pelvic cavity and the inferior true pelvic cavity.

### 3.3 Walls of trunk cavities

From the description above it is apparent that the boundaries of an anatomical volume can be virtual, for example, thoracic inlet, or structural, for example, thoracic diaphragm, and when an anatomical structure forms the vertical boundary this is often described as a ‘wall’.

The FMA defines the trunk body wall as a ‘subdivision of trunk that consists of those organs that separate the body cavity from the body's exterior; together with the body cavity and its contents, the body wall constitutes the trunk’.

The constituent layers of the trunk wall vary between, and within, different cavities: The inner wall boundary is consistent, but the extent to which more superficial layers are included as ‘part of the wall’ is dependent on the area and the walls of the three main volumes are considered below.

#### 3.3.1 Chest wall structure

The wall of thorax is defined in FMA as a ‘Heterogeneous cluster which surrounds the thoracic cavity and its content; and which includes the ribcage, muscle group of thoracic wall and costal pleura’: thus, the thoracic wall is entirely comprised of a skeletal frame, and excludes the overlying integument. The ‘chest wall’ is a broader concept than the thoracic wall and is defined in FMA as the ‘subdivision of thorax which includes all structures from the skin to the costal pleura’, for example, it includes the wall of thorax, the superficial chest wall, lateral chest wall and anterior chest wall including the integument.

The FMA also segments the wall of thorax into the:

- Anterior, right and left lateral thoracic region, which includes the costal pleura, endothoracic fascia, intercostal muscles, ribs and costal cartilages, transversus thoracis and subcostal muscle layers (but not the integument); and
- Posterior thoracic wall region, which is delineated as the section posterior to the mid‐axillary line and is constituted by the costal pleura, endothoracic fascia and thoracic vertebrae.

In keeping with the above definitions, the chest wall may be considered as having an anterior, lateral and posterior region. The most medial section of the posterior thoracic region contributes to the thoracic region of back that also includes the thoracic vertebral column and the erector spinae muscles.

#### 3.3.2 Wall of abdominopelvic segment of trunk

The wall of the abdominopelvic cavity has two regional parts: the wall of abdomen proper cavity and the wall of pelvic cavity. In addition, the upper abdominal cavity gains protection from the lower six ribs and their cartilages, even though these structures are technically part of the thoracic wall.

**FIGURE 3:**
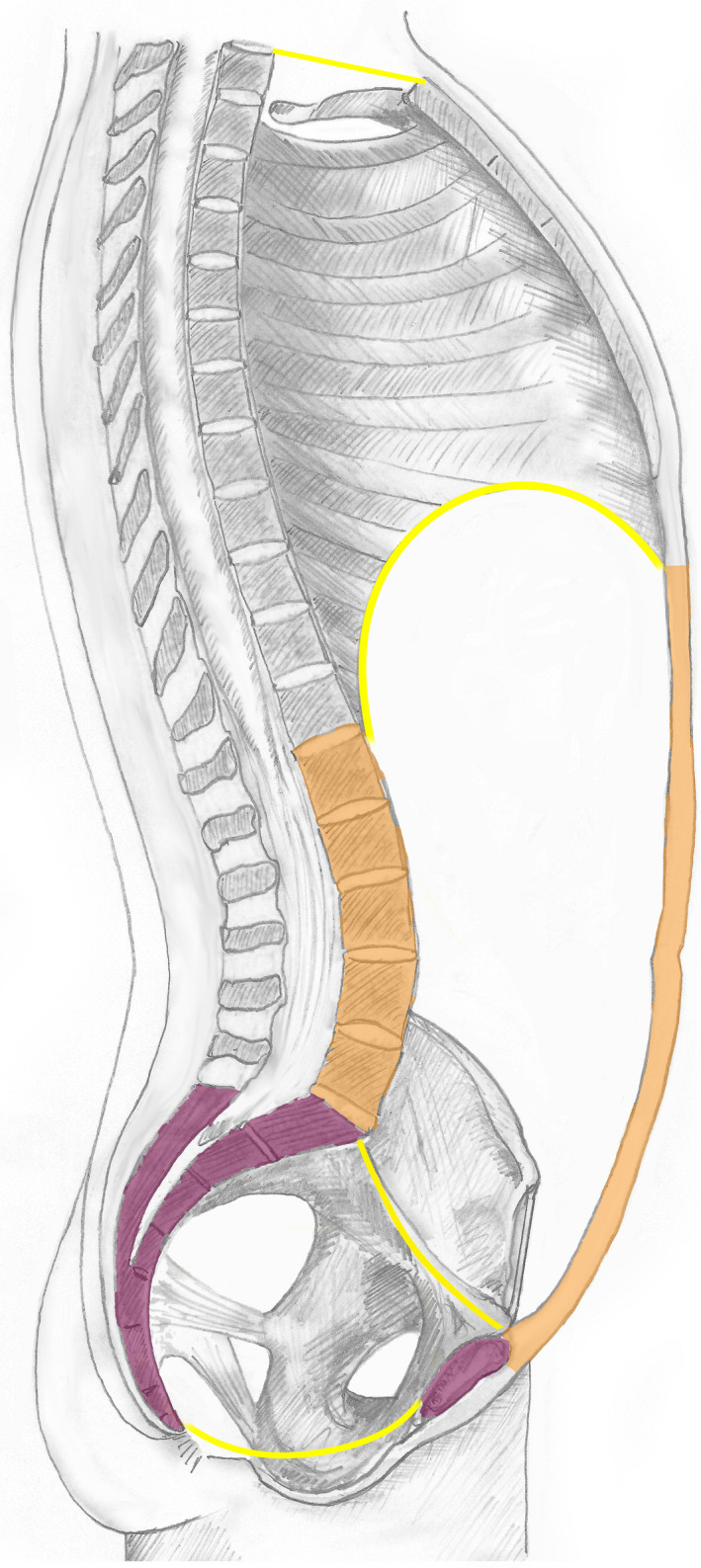
Walls of the trunk cavities. The anterior and posterior walls of the ‘abdomen proper cavity’ are illustrated in amber and the walls of ‘cavity of true pelvis’ in purple. The thoracic diaphragm forms the superior boundary of the abdominopelvic cavity and the pelvic diaphragm delineates the inferior boundary. The superior pelvic aperture is the virtual plane that separates the ‘abdomen proper cavity’ from the ‘cavity of true pelvis’

The anterior and posterior walls of the ‘abdomen proper cavity’ are illustrated in amber and the wall of ‘true pelvic cavity’ in purple in Figure 3, which in combination form the abdominopelvic wall.

#### 3.3.3 Structure of wall of abdominal proper segment of trunk

The wall of abdomen proper cavity is comprised of an anterior and posterior component:

The anterior abdominal wall includes the anterior and lateral sections of the abdominal wall, delineated by a virtual vertical mid‐axillary boundary line from the posterior component of the abdominal wall; it is bounded superiorly by the xiphisternal joint and the costal margins; inferiorly the symphysis pubis, the inguinal ligament and the iliac crests (Figure 1). Thus, the ‘inguinal region’ is only partly located within the lower and lateral abdominal region; the structures below and external to the inguinal ligament are a component of the lower limb.

The anterior abdominal wall is comprised of a number of layers including the:

- Skin and superficial fascia of anterior part of abdomen
- Musculature of anterior abdominal wall (comprising the external oblique, transversus abdominis, rectus abdominis, internal oblique and pyramidalis)
- Anterior part of abdominal peritoneum.

The definition of the posterior abdominal wall is contentious but the following structures constitute the immediate deep components, which can be considered as the ‘posterior wall of abdomen proper’:

- Posterior abdominal wall musculature (quadratus lumborum, psoas major and psoas minor)
- Crus of lumbar part of diaphragm
- Lumbar vertebrae bodies and transverse processes
- Posterior part of abdominal peritoneum
- The abdominal wall including the transversus abdominus, internal and external oblique muscles posterior to the mid‐axillary line.

**FIGURE 4:**
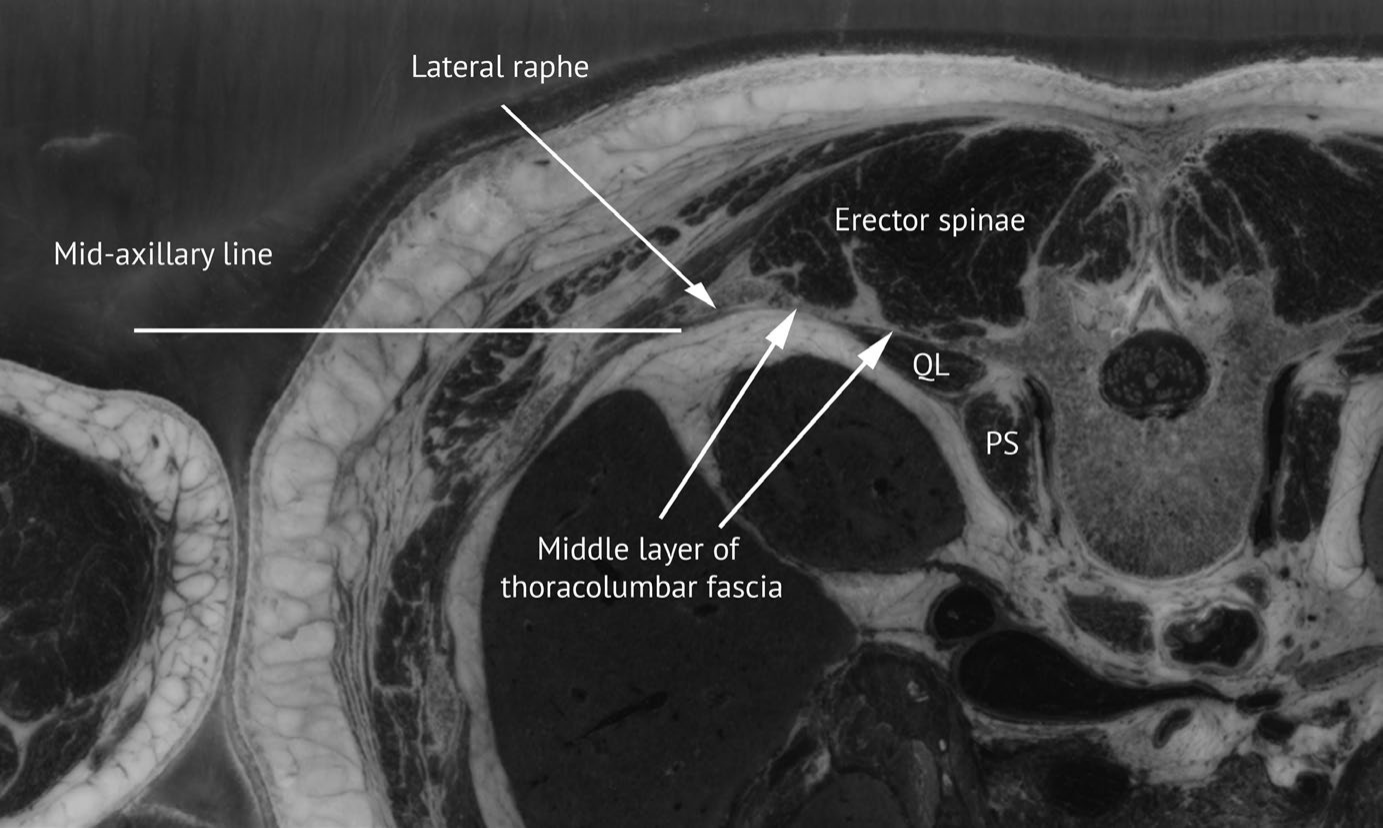
Posterior wall of abdomen proper. Cross‐section of the lumbar region of abdomen illustrating the posterior wall of abdomen proper (PWAP) http://snomed.info/id/827003001 consisting of the quadratus lumborum [QL], psoas muscles (only the psoas major [PS] shown in this cross section) and the body and transverse process of the lumbar vertebra. The posterior boundary of the PWAP is constituted by the middle layer of the thoracolumbar fascia and the lateral raphe, separating it from the lumbar region of back and its constitutional parts including the erector spinae. The mid‐axillary line separates the PWAP from the anterior and lateral abdominal wall. The cross section is from the Visible Human Project (slice afv1588c, courtesy of the U.S. National Library of Medicine)

Some sources include additional structures within the posterior abdominal wall that are located more posteriorly (superficial) than the lumbar vertebral bodies, but the presented definition is in accordance with Gray's anatomy (Stringer et al., 2015:1033) that states that the posterior abdominal wall (proper) comprises of ‘five lumbar vertebrae and their intervening intervertebral discs...[and] the muscles of the posterior abdominal wall’, that is, it excludes the more superficial layers of muscle and skin of the back. The definition is also in general agreement with the FMA. The quadratus lumborum, psoas, lumbar vertebral bodies and their transverse processes all fulfil the criteria of containment and protection as described above. These structures are separated from erector spinae by the middle layer of the thoracolumbar fascia (medially attached to the transverse process of the vertebra), which along with the lateral raphae constitute the posterior boundary of the posterior wall of abdomen proper (Figure 4).

#### 3.3.4 Pelvic wall structure

The wall of the pelvis is skeletal and is comprised of the bones, joints, ligaments of the pelvis and associated muscles (piriformis and obturator internus). It forms part of the wall of abdominopelvic cavity and the pelvic region of trunk. The wall of the entire pelvis includes the ilia above the superior pelvic aperture that forms the boundary of the false pelvic cavity; the wall of the true pelvic cavity lies below this boundary and consists of:

- Anteroinferiorly, the pubic bones, their rami and the symphysis pubis;
- Posteriorly, the sacrum and coccyx;
- Laterally on each side, its margins are the smooth quadrangular pelvic aspect of the fused ilium and ischium and the ligaments that interconnect these bones, and the muscles that line their inner surfaces below the superior pelvic aperture (piriformis and obturator internus) and
- Inferiorly, the pelvic floor forms the junction with the pelvic wall.

#### 3.3.5 Pelvic floor, pelvic diaphragm and perineum

In clinical practice and in some literature, the pelvic floor and pelvic diaphragm are frequently used interchangeably and FMA also utilises pelvic floor as a ‘synonym’ of pelvic diaphragm: however, technically the pelvic floor is a broader, less specific concept than the pelvic diaphragm. Gray's anatomy states that the pelvic ‘diaphragm’ consists of the levator ani (pubococcygeus, iliococcygeus and puborectalis); the ischiococcygeus, and in combination with the two fascial layers and this structure delineates the lower limit of the true pelvis (Delancey, 2015:1221‐22). The female pelvic ‘floor’ also includes the ligamentous supports of the cervix, and the pelvic and urogenital diaphragms (Collins et al., 2015).

The perineum is defined as a structure bounded anteriorly by the pubic symphysis and its arcuate ligament; posteriorly by the coccyx; anterolaterally by the ischiopubic rami and the ischial tuberosities; and posterolaterally by the sacrotuberous ligaments. The deep limit of the perineum is the inferior surface of the pelvic diaphragm, and its superficial limit is the skin that is continuous with that over the medial aspect of the thighs and the lower abdominal wall. As a consequence of this the female perineum subsumes the external genitalia of the vulva, but the male perineum excludes the entire external genitalia (penis, scrotal and testis structures).

It is also worth noting that the wall of pelvis excludes the integument and mons pubis; but these are constituents of the pelvic segment of trunk and pelvic region (Figures 2 and 9).

### 3.4 Regional segments of the trunk

In many scenarios, the trunk may be considered to be composed of three regional segments (thoracic, abdominal and pelvic) and, although they are sometimes referred to in the literature, there are no existing standard definitions.

#### 3.4.1 Thoracic segment of trunk

**FIGURE 5:**
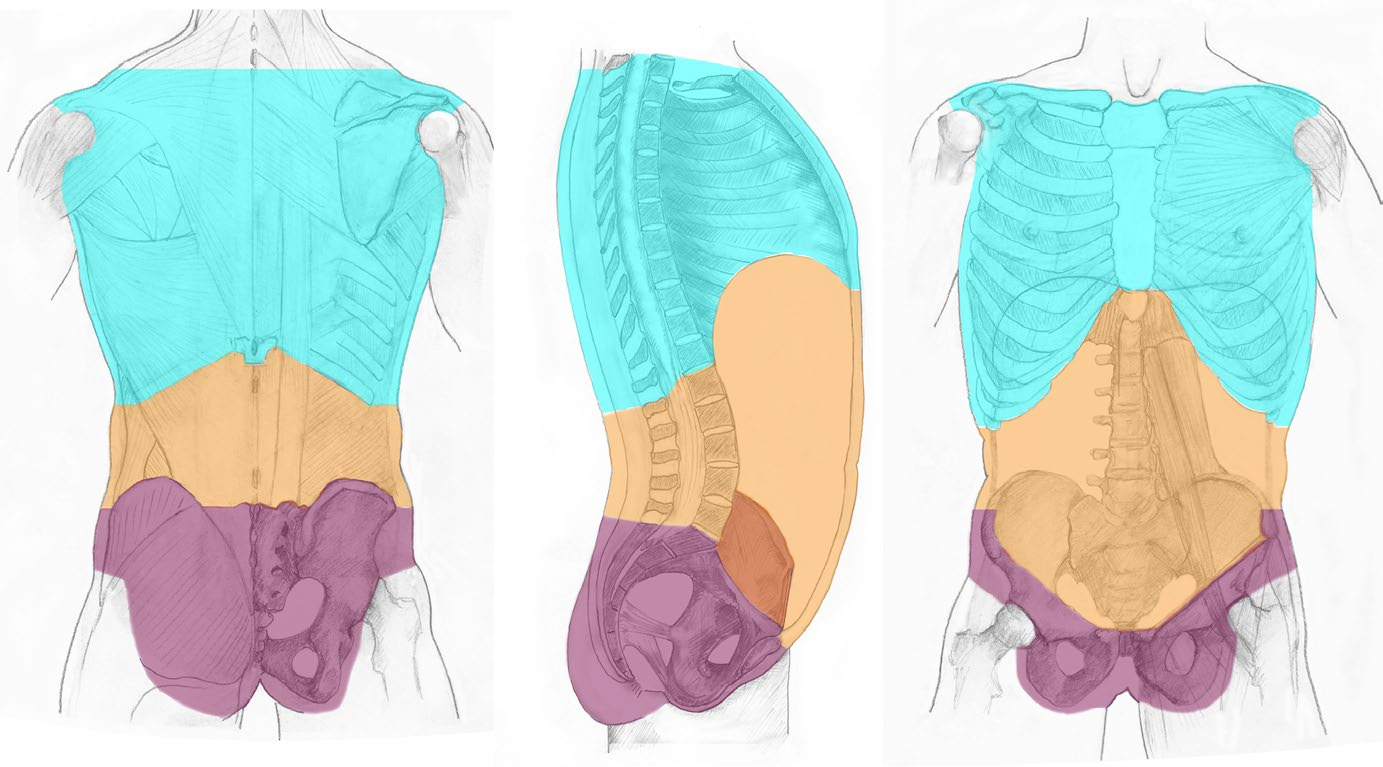
Regional segments of the trunk. The illustration identifies the thoracic segment of trunk http://snomed.info/id/67734004 (cyan), abdomen proper segment of trunk http://snomed.info/id/818985005 (amber) and the pelvic segment of trunk http://snomed.info/id/609617007 (purple)

This segment relates to the volume of the trunk that is delineated by, and includes superiorly the thoracic inlet: inferiorly the thoracic diaphragm; and the chest wall. It incorporates the thoracic cavity, contents and wall, including the thoracic vertebral column and all the overlying muscles, skin and subcutaneous tissue (Figure 5). This definition is in harmony with FMA, which defines the thoracic segment of trunk as being a ‘subdivision of trunk which has as its parts the thorax and the back of thorax’. The back of thorax is described as being constituted of ‘some complete set of vertebral arches (T1‐T12) and anatomical entities located posterior to them’, and the thorax as ‘a subdivision of front of trunk, each instance of which has as its constitutional part some complete set of thoracic vertebral bodies (T1‐T12) and some ribcage’.

#### 3.4.2 Abdominopelvic segment of trunk

This segment relates to the volume of the trunk that is bounded by and includes: superiorly the thoracic diaphragm and inferiorly the perineum and external genitalia. The volume includes the entire transverse thickness of the body over the longitudinal extent between these upper (superior) and lower (inferior) boundaries including the overlying muscles, skin and subcutaneous tissue. The segment includes the abdominopelvic cavity, contents and wall including the posterior lumbar region; the volume of the true and false pelvic cavities; the bony pelvis and pelvic wall; the entire perineum and external genitalia including skin and subcutaneous tissue.

This trunk segment can be subdivided into the ‘abdomen proper segment of trunk’ and the pelvic segment of trunk, and are shown, respectively, in amber and purple in Figure 5.

#### 3.4.3 Abdomen proper segment of trunk (Structure of abdominopelvic segment excluding true pelvic segment of trunk)

This segment relates to the volume of the trunk that is delineated superiorly by, and includes the thoracic diaphragm, and inferiorly by the superior pelvic aperture. It incorporates the abdominal proper cavity, contents and wall including the lumbar vertebral column and all the overlying muscles, skin and subcutaneous tissue. It consequently includes the intra‐abdominal proper structures (both the abdomen proper cavity and its contents), the anterior and abdominal wall and the posterior lumbar region. Note, that the entire bony pelvis, although forming part of the boundary, is excluded from the abdomen proper segment of trunk, but is included as part of the pelvic segment of trunk (Figure 5).

#### 3.4.4 Structure of pelvic segment of trunk

This segment relates to the volume of the trunk that is bounded by and includes: superiorly the boundary of the false pelvis, which is an artificial plane from the symphysis pubis to the superior iliac crests (in front it is incomplete, presenting a wide interval between the anterior borders of the ilia); and inferiorly the perineum and external genitalia. The segment includes the volume of the true and false pelvic cavities including the bony pelvis and pelvic wall and the entire perineum and external genitalia. The volume includes the entire transverse thickness of the body over the longitudinal extent between these upper (superior) and lower (inferior) boundaries including the overlying muscles, skin and subcutaneous tissue. The entire bony pelvis is included within the pelvic segment (but is excluded from the abdomen proper segment of trunk) (Figure 5). It is note worthy that the pelvic segment of trunk and the abdomen proper segment of trunk both include the volume of the false pelvis, that is, they are not disjoint; this is because the false pelvis contains structures such as the iliac vessels that are generally regarded clinically to be in the ‘pelvic region’. An additional notion of the ‘true pelvic segment of trunk’ that excludes the cavity and contents of the false pelvis was recognised, but no current clinical requirement has been identified.

### 3.5 Cross‐sectional segments of the trunk

**FIGURE 6:**
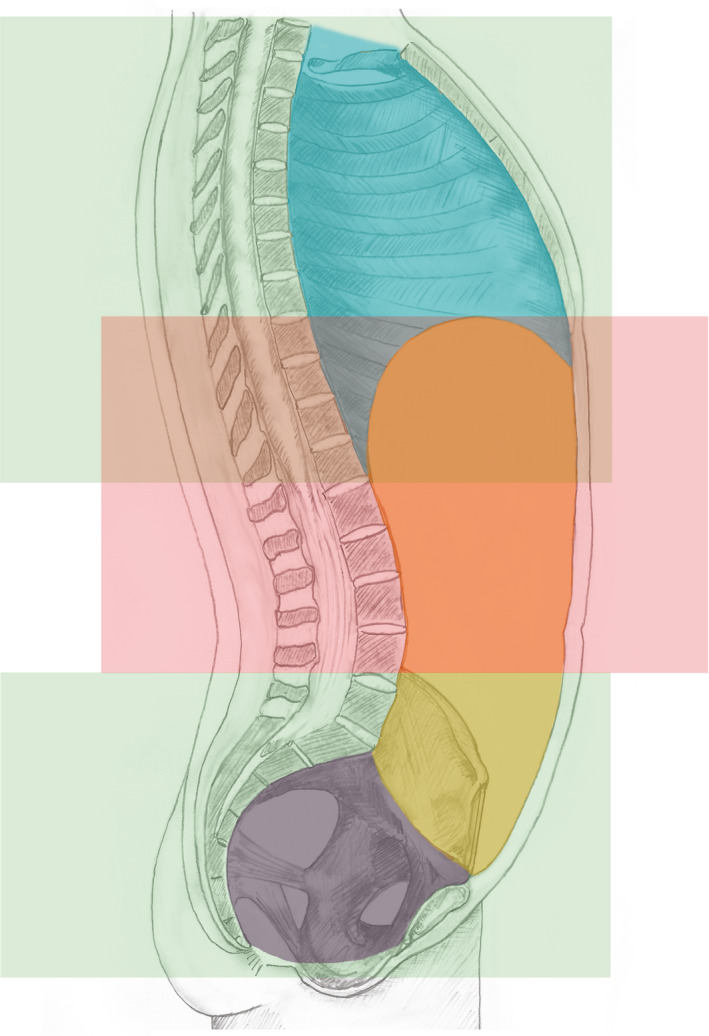
Cross‐sectional segments of the trunk. The segments relate to the projectional volume as perceived by transmissive or emissive imaging and the volume includes the entire transverse thickness of the body over the longitudinal extent between the superior (upper) and inferior (lower) boundaries including the skin and subcutaneous tissue and surrounding musculoskeletal structures. The thoracic cross‐section of trunk http://snomed.info/id/816094009 (upper green volume) extends from the boundary of first thoracic vertebra (T1) to a virtual horizontal plane at the level of the lower boundary of the twelfth thoracic vertebra (T12/L1 junction); it overlaps with the cross‐sectional segment of abdomen http://snomed.info/id/818981001 (pink), which extends from the level of T8/T9 to the superior boundary of the iliac crest. The pelvic cross‐sectional segment of trunk http://snomed.info/id/816092008 (lower green volume) extends from a virtual horizontal plane at a level traversing the superior boundary of the iliac crest to the perineum and includes part but not necessarily the entire external genitalia

In diagnostic radiology, the term ‘abdomen’ is used in the context of imaging procedures, which are cross‐sectional or projectional (transmissive) or emissive. For cross‐sectional procedures especially, such as CT, MRI and Positron Emission Tomography (PET), a procedure of the abdomen is distinguished from a procedure of the pelvis (below) or chest (above), and the entire transverse thickness of the body over that longitudinal extent is examined. For example, the ‘abdominal cross‐sectional segment of trunk’, that is, the radiological ‘abdomen’, is full thickness (segment of trunk, not confined to the front, or the cavity and including the skin of the front and back). Similarly concepts are also required for the ‘thoracic cross‐sectional segment of trunk’ and ‘pelvic cross‐sectional segment of trunk’ (Figure 6).

The boundaries of these regions do not adhere to traditional anatomical borders, partly because the segments are horizontal transverse sections, comparable to virtual anatomical planes, for example, the thoracic inlet. The following section illustrates these cross‐sectional regional concepts, including those established within radiology. It is critical to reiterate that the ‘cross‐sectional segments’ include all structures between the planes identified, that is, the regions include the skin and subcutaneous tissue and surrounding musculature as well as the cavity wall and contents; furthermore, the segments may overlap (are not necessarily disjoint).

#### 3.5.1 Thoracic cross‐sectional segment of trunk

This cross‐sectional segment is bounded superiorly by a virtual horizontal plane at the level of the thoracic inlet (upper boundary of first thoracic vertebra (T1) and inferiorly by a virtual horizontal plane at the level of the lower boundary of the twelfth thoracic vertebra (T12/L1 junction). The segment relates to the projectional volume as perceived by transmissive or emissive imaging and consequently the volume includes the entire transverse thickness of the body over the longitudinal extent between the superior (upper) and inferior (lower) boundaries including the skin and subcutaneous tissue and surrounding musculoskeletal structures; and subsumes the entire thoracic cavity and also part of the upper abdominal volume above the virtual T12/L1 vertebra plane level (upper volume highlighted green in Figure 6). This segment also overlaps with the abdominal cross‐sectional segment of trunk at level of T8/T9 to T12/L1.

#### 3.5.2 Abdominopelvic cross‐sectional segment of trunk

This cross‐sectional segment is bounded superiorly by a virtual horizontal plane at the level of the junction between the eighth and ninth thoracic vertebrae (T8/T9) and inferiorly extends to the perineum, including the volume of the true and false pelvic cavities and part but not necessarily the entire external genitalia. The segment relates to the projectional volume as perceived by transmissive or emissive imaging (although does not account for the angulation of a diverging X‐ray beam from a point source) and its volume includes the entire transverse thickness of the body over the longitudinal extent between the superior (upper) and inferior (lower) boundaries including the skin and subcutaneous tissue and surrounding musculoskeletal structures; and subsumes the entire abdomen proper, the majority of the pelvic region and part of the thoracic volume below the level of T8/T9. This segment is a combination of the abdominal and pelvic cross‐sectional segments of trunk (highlighted pink and lower volume green in Figure 6).

#### 3.5.3 Abdominal cross‐sectional segment of trunk

This cross‐sectional segment is bounded superiorly by a virtual horizontal plane at the level of the junction T8/T9 and inferiorly by a virtual horizontal plane traversing the superior boundary of the iliac crest at the level of the intercristal line (also termed Jacoby's or Tuffler's line), which is approximately at the level of L4 in men and L5 in women. The segment relates to the projectional volume as perceived by transmissive or emissive imaging and this volume includes the entire transverse thickness of the body over the longitudinal extent between the superior (upper) and inferior (lower) boundaries including the skin and subcutaneous tissue and surrounding musculoskeletal structures; and subsumes the abdominal proper down to the level of L4 or L5 and also part of the thoracic volume below the T8/T9 level (highlighted pink in Figure 6).

#### 3.5.4 Pelvic cross‐sectional segment of trunk

This cross‐sectional segment is bounded superiorly by a virtual horizontal plane at a level traversing the superior boundary of the iliac crest (approximately at the level of L4 in men and L5 in women), and inferiorly extending to the perineum and including part but not necessarily the entire external genitalia. The segment relates to the projectional volume as perceived by transmissive or emissive imaging and includes the entire transverse thickness of the body over the longitudinal extent between the superior (upper) and inferior (lower) boundaries including the skin and subcutaneous tissue and surrounding musculoskeletal structures; and subsumes the entire volume of the true and false pelvic cavities; part of the lower abdominal volume below the level of the virtual superior boundary and a volume below the pelvic diaphragm which constitutes part of the perineum and part, but not necessarily all, of the external genitalia (lower volume highlighted green in Figure 6). A synopsis of the main relationships of these defined structures of cross sections and segments of trunk are illustrated in Figure 10.

### 3.6 Content of trunk cavities

In SNOMED CT, the notions of intra‐thoracic structure, intra‐abdominopelvic structure, intra‐abdominal proper structure and intra‐pelvic structure of true pelvis, all subsume both the cavity and contents. The modelling supports any conditions or procedures that involve the cavity, content or both.

For example, the ‘intra‐abdominopelvic structure’ (structure of abdominopelvic cavity and/or content) comprises the space and content within the boundaries of the abdominopelvic cavity but excludes the walls that delineate the space: the defined boundary wall structures consist of superiorly the thoracic diaphragm; inferiorly the pelvic diaphragm; anteriorly the anterior abdominal wall (which includes the lateral abdominal wall) and posteriorly the ‘posterior wall of abdomen proper’.

The intra‐pelvic structure of true pelvis (structure of cavity and/or content of true pelvis) consists of the cavity and contents of the true pelvis, which is bounded by, but excludes, the pelvic wall and inferiorly the pelvic diaphragm and subsumes only anatomical structures entirely located within the cavity of the true pelvis, that is, the contents within the cavity above the pelvic diaphragm and below the superior pelvic aperture such as the urinary bladder (Figure 10). The contents is dependent on gender as illustrated below by listing conjoint significant anatomical structures first, followed by those that are gender specific:

**Table:** 

Male	Female
Urinary bladder	Urinary bladder
Retropubic space	Retropubic space
Presacral space	Presacral space
Sigmoid colon	Sigmoid colon
Rectum	Rectum
Pelvic portion of ureter	Pelvic portion of ureter
Puboprostatic ligament	Uterus
Prostate	Ovary
Seminal vesicles	Fallopian tubes

The identification of structures within such cavities, regional and cross‐sectional volumes of the trunk requires careful consideration, as from a formal modelling perspective, the entire structure has to be contained within the wall or boundary of the volume to be regarded heuristically as a constitutional part, or assumptions will be flawed.

For example, some literature regards the large bowel as being within the abdominopelvic cavity, and selected coding schemas consider the anal canal to be part of the large intestine, for example, ICD‐11 (XA39S6). From an ontological perspective, this is incorrect because the anal canal is distal to the anorectal ring at the level of the pelvic diaphragm, which marks the inferior boundary of the abdominopelvic cavity; the anal canal, therefore, is not entirely contained within the abdominopelvic cavity and as a consequence neither is the entire large intestine. Another example relates to the distal segment of the descending colon that extends below the iliac crest into the false pelvis, historically this segment was known as the ‘iliac colon’: although the terminology has fallen out of common usage, the distinction is, however, valuable for modelling, as the ‘iliac colon’ can be considered to be an intra‐abdominopelvic proper structure and also within the pelvic segment of trunk (and ‘pelvic region’, see below).

### 3.7 Complex regions of the trunk

Clinical notions of truncal regions often relate to combinations of anatomical structures into more complex conglomerations. For example, the term ‘abdominopelvic region’ is frequently used but it is ambiguous with respect to its boundaries and contents, and in common with a number of other regional notations, there is no agreed definition. In this study we describe the most frequently used clinical conglomerations and sub‐regions of the trunk, clearly defined by their boundaries to avoid such ambiguity.

#### 3.7.1 Structure of abdominopelvic cavity and/or content of abdominopelvic cavity and/or anterior abdominal wall (Abdomen)

**FIGURE 7:**
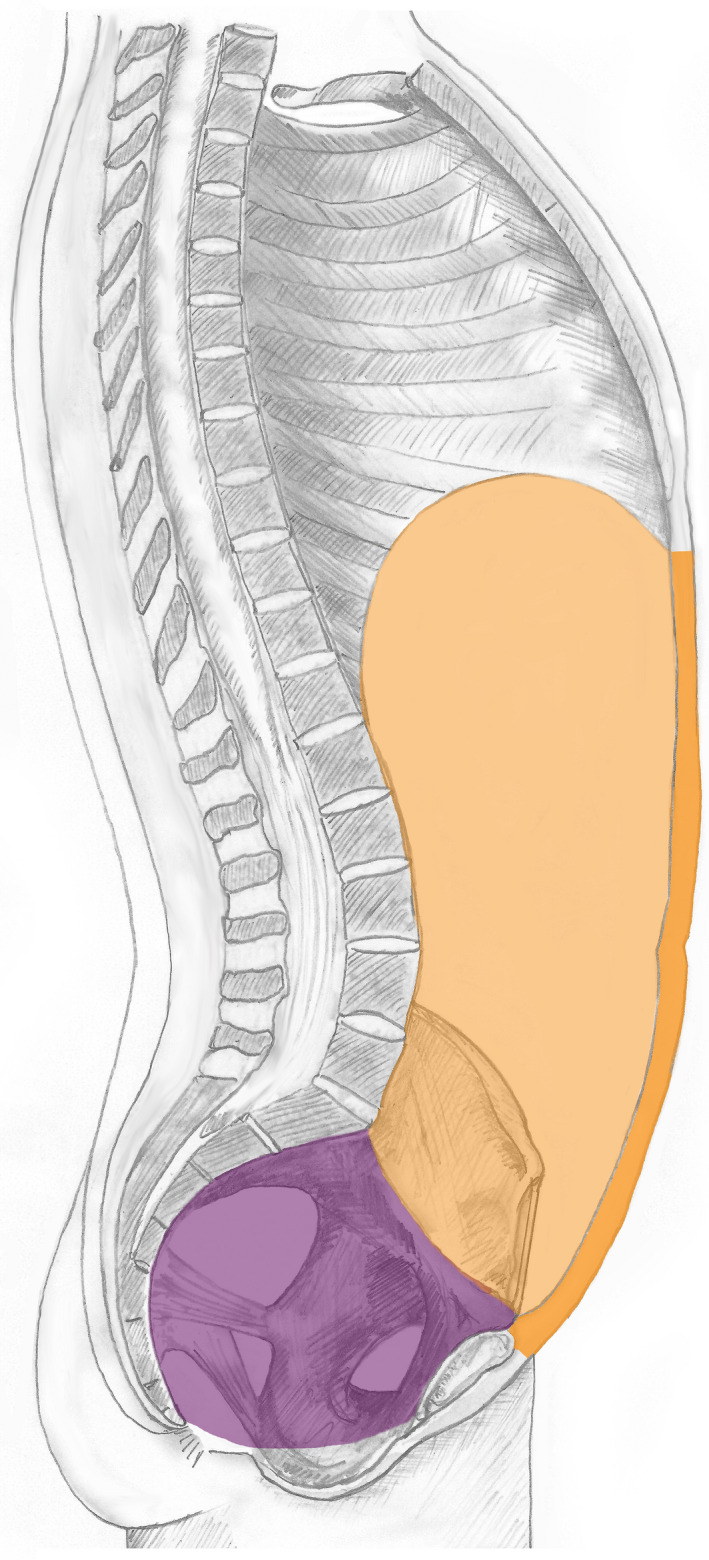
The abdomen. The illustration summarises the most common clinical interpretation of the meaning of ‘abdomen’ that includes the abdominopelvic cavity and/or content of abdominopelvic cavity and/or anterior abdominal wall http://snomed.info/id/818983003

This is considered the most commonly used clinical variant of ‘abdomen’ and relates to the space and contents within the abdominopelvic cavity plus the anterior abdominal wall. The volume is bounded by, but excludes: superiorly the thoracic diaphragm; inferiorly the pelvic diaphragm and posteriorly the posterior wall of abdomen proper; the pelvic component consists of the cavity and contents of the true pelvis, which is bounded by, but excludes, the pelvic wall and pelvic diaphragm. This variant is illustrated in Figure 7 and within SNOMED CT has been exclusively allocated the term ‘Abdomen’ (see below).

#### 3.7.2 Structure of abdominopelvic cavity and/or intra‐abdominopelvic content and/or anterior abdominal wall excluding intra‐pelvic structure of true pelvis (Abdomen proper)

**FIGURE 8:**
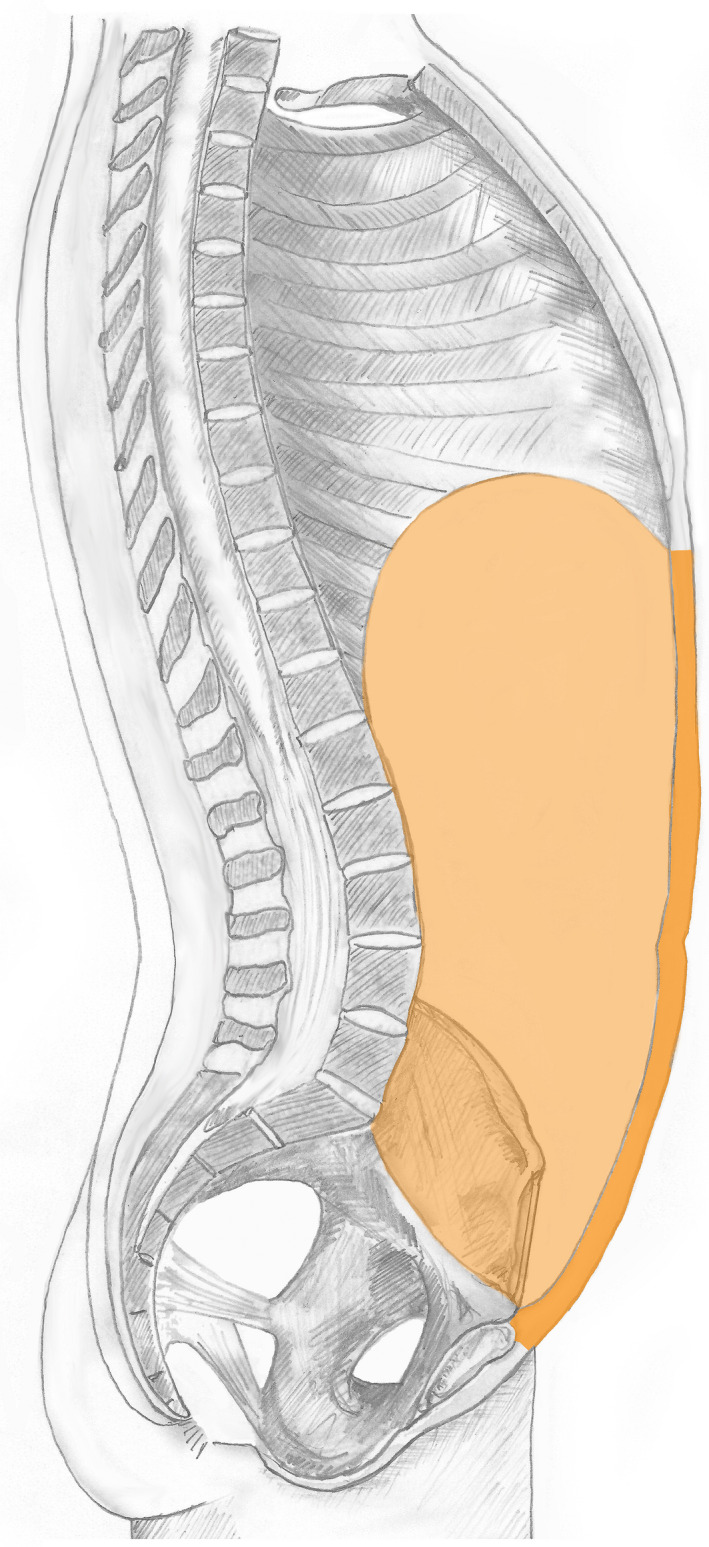
The abdomen proper. The illustration summarises the more limited meaning of ‘abdomen proper’ that includes the anterior abdominal wall and intra‐abdominopelvic structures but excluding the cavity and content of the true pelvis http://snomed.info/id/818984009

This is a clinical variant of the abdomen relating to the ‘abdomen proper’ which subsumes the space and contents within the abdominopelvic cavity, but excluding the cavity and content of the true pelvis, plus the anterior abdominal wall. This space and content are bounded by, but exclude: superiorly the thoracic diaphragm; inferiorly the superior pelvic aperture and posteriorly the posterior wall of abdomen proper. Anteriorly this volume is bounded and includes the anterior abdominal (including the lateral abdominal wall). This variant is illustrated in Figure 8 and within SNOMED CT has been exclusively allocated the term ‘Abdomen proper’ (see below).

#### 3.7.3 Structure of pelvis (Pelvic region)

SNOMED CT includes the notion of ‘structure of pelvis’, which is a complex clinical regional concept that incorporates the entire bony pelvis, the true and false cavities and their contents, and inferiorly it is bounded and includes the pelvic diaphragm. The structure is comprised of the complete pelvic wall, sacrococcygeal region and includes the overlying skin and subcutaneous tissue including the mons pubis, but excludes the perineum and external genitalia.

**FIGURE 9:**
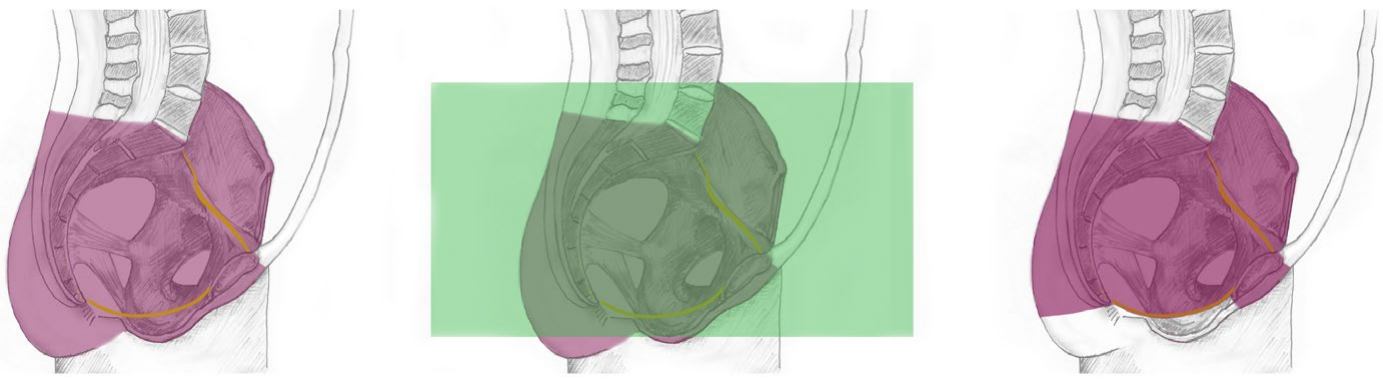
Comparison volumes of the pelvic segment of trunk, pelvic cross‐sectional segment of trunk and the pelvic structure (pelvic region). The illustration compares the subtle differences between the pelvic segment of trunk http://snomed.info/id/609617007 (left) that includes the true and false pelvis including the overlying skin and subcutaneous tissue, the entire perineum and external genitalia; the pelvic cross‐sectional segment of trunk http://snomed.info/id/816092008 (middle, green volume), which subsumes the entire cross‐sectional portion below the superior boundary of the iliac crest but not necessarily the entire external genitalia; and the pelvic structure (pelvic region) http://snomed.info/id/12921003 (right), which is similar to the pelvic segment of trunk apart from inferiorly it is bounded by and includes the pelvic diaphragm, but excludes the perineum and external genitalia

The ‘pelvic region’ is part of both the pelvic cross‐section segment of trunk and the pelvic segment of trunk, which also both subsume the perineum; but only the pelvic segment of trunk necessarily includes the entire external genitalia (Figure 9).

**FIGURE 10:**
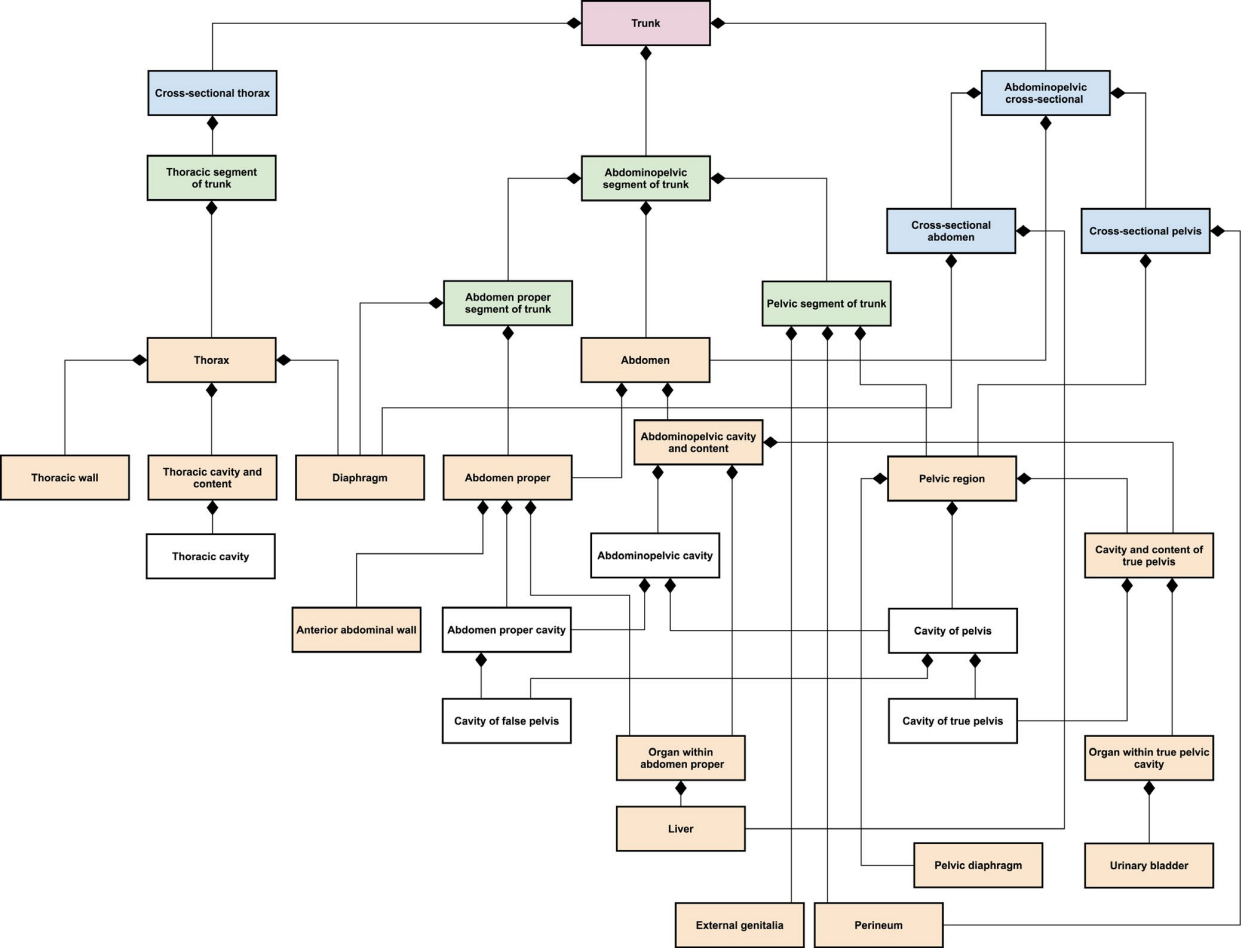
Partonomic class diagram of anatomical structures and regions of the trunk. The illustration demonstrates merynomic relationships of key volumes of the trunk using UML notation. The concepts are labelled using shortened terms to aid readability, for example, Abdomen is used for Structure of abdominopelvic cavity and/or content of abdominopelvic cavity and/or anterior abdominal wall. The figure is a synopsis of the most important relationships and does not include all intermediate relationships; the full hierarchy is available by visiting https://browser.ihtsdotools.org/?

The merynomic relationships of these complex regions and other key volumes of the trunk are illustrated in Figure 10 using Unified Modelling Language (UML) notation (UML, 2011); this details the most important concepts discussed and to assist clarity does not include all intermediate relationships, for example, the ‘structure of true pelvis’ is not displayed as an intermediate concept between ‘cavity and content of true pelvis’ and the ‘pelvic region’ (the full hierarchical view is available by visiting https://browser.ihtsdotools.org/?).

### 3.8 Implementation and quality assurance of clinical regions

Following focused consultation and feedback from clinical and informatics experts on the preceding proposal, the concepts and relationships documented above were integrated into SNOMED CT. The impact of this integration was evaluated by reviewing the classification changes in the dependent disorder and procedure hierarchies (modelled with the associated anatomical concepts). This process identified reliant concepts that had incorrect new placements or inadvertently lost relationships; these were due either to errors in hierarchical placement of subordinate anatomical concepts or suboptimal definitions of the dependent finding and procedures. These errors were corrected consecutively and their impact on the hierarchies scrutinised in an iterative process.

Expert responses also agreed that the proposed model should bring greater clarity and shared understanding of the meaning and use of the identified clinical regional concepts; they also endorsed the significance attributed to the ambiguity and variability of using the term ‘abdomen’ and recommended greater stakeholder engagement. It was also acknowledged that it would be inappropriate to prohibit the use of the term ‘abdomen’ within a terminology (and impossible in clinical practice), and two possible solutions to this issue were considered:

Firstly, one could assign the term abdomen as a ‘shared synonym’ to multiple concepts (polysemy). This *en face* is an attractive solution but its successful implementation is dependent, and places the onus, on software developers to avoid bias by displaying the polysemy to users clearly and consistently to prompt them to choose the correct concept from the choice of unambiguous FSNs of the near‐variant concepts.

Secondly, one could unequivocally designate the term ‘abdomen’ to a single concept and use more explicit concatenated terms for the other near‐variant concepts. This is a more simplistic solution but has the disadvantage of allocating one notion as the default scenario.

Following a call for wider consultation with interested parties and studying responses, SNOMED International decided to implement the second option of allocating the term ‘abdomen’ to the most common clinical situation: An argument for this more dogmatic approach was that the use of the ‘preferred synonyms’ of near variant concepts would prompt users of the terminology to explicitly choose the most suitable concept (and might also prompt their use in clinical practice and documentation).

In summary, three important complex regional clinical variants were identified during the work for which the following ‘preferred synonyms’ have been allocated:

- Intra‐abdominopelvic structure and/or anterior abdominal wall – synonym: *Abdomen*
- Intra‐abdominopelvic structure and/or anterior abdominal wall, excluding intra‐pelvic structure of true pelvis – synonym: *Abdomen proper*
- Structure of abdominal cross‐sectional segment of trunk – synonym: *Cross‐sectional abdomen*

These complex regional concepts and other clinical important variants are summarised in Table 2.

**TABLE 2:** Summary of regional anatomical concepts of the trunk

SCTID	Term & FSN	Definition
	SEGMENT	
67734004	Thoracic segment of trunk Structure of thoracic segment of trunk (body structure)	This segment relates to the volume of the trunk that is bounded by and includes: superiorly the thoracic inlet; inferiorly the thoracic diaphragm; posteriorly the spinal column and back of the thorax; and laterally the chest wall: it contains the thoracic cavity and contents, and includes all the overlying muscles, skin and subcutaneous tissue.
818985005	Abdomen proper segment of trunk Structure of abdominopelvic segment excluding true pelvic segment of trunk (body structure)	This segment relates to the volume of the trunk that is bounded superiorly by and includes: the thoracic diaphragm and inferiorly by the superior pelvic aperture. It incorporates the abdominal proper cavity (which includes the volume of the false pelvis), contents and wall including the lumbar vertebral column and all the overlying muscles, skin and subcutaneous tissue. It consequently includes the intra‐abdomen proper structure, the anterior abdominal wall and the posterior lumbar region. Note, the entire bony pelvis although forming part of the boundary is excluded from the abdomen proper segment of trunk (but is included as part of the pelvic segment of trunk).
609617007	Structure of pelvic segment of trunk Structure of pelvic segment of trunk (body structure)	This segment relates to the volume of the trunk that is bounded by and includes: superiorly the boundary of the false pelvis, which is an artificial plane from the symphysis pubis to the superior iliac crests (in front it is incomplete, presenting a wide interval between the anterior borders of the ilia); and inferiorly the perineum and external genitalia. The volume includes the entire transverse thickness of the body over the longitudinal extent between these upper (superior) and lower (inferior) boundaries including the overlying muscles, skin and subcutaneous tissue. The segment includes the volume of the true and false pelvic cavities, including the bony pelvis and pelvic wall and the entire perineum and external genitalia. Note, the entire bony pelvis is included within the pelvic segment (but is excluded from the abdomen proper segment of trunk); however, the pelvic segment of trunk and the abdomen proper segment of trunk both include the volume of the false pelvis.
	CROSS SECTION	
816094009	Cross‐sectional thorax Structure of thoracic cross‐sectional segment of trunk (body structure)	This cross‐sectional segment is bounded superiorly by a virtual horizontal plane at the level of the thoracic inlet (upper boundary of first thoracic (T1) vertebra) and inferiorly by a virtual horizontal plane at the level of the lower boundary of the twelfth thoracic (T12) vertebra. The segment, therefore, includes the entire thoracic cavity but also part of the upper abdominal volume above the virtual plane at the T12 vertebra. The volume includes the entire transverse thickness of the body over the longitudinal extent between the superior (upper) and inferior (lower) boundaries including the skin and subcutaneous tissue. The segment relates to the cross‐sectional or projectional volume as perceived by transmissive or emissive imaging.
818981001	Cross‐sectional abdomen Structure of abdominal cross‐sectional segment of trunk (body structure)	This cross‐sectional segment is bounded superiorly by a virtual horizontal plane at the level of the junction between T8 and T9 (and, thus, also includes part of the thoracic volume below this level); and inferiorly by a virtual horizontal plane at the level of the plane traversing the superior boundary of the iliac crest. The volume includes the entire transverse thickness of the body over the longitudinal extent between the superior (upper) and inferior (lower) boundaries including the skin and subcutaneous tissue. The segment relates to the cross‐sectional or projectional volume as perceived by transmissive or emissive imaging.
816092008	Cross‐sectional pelvis Structure of pelvic cross‐sectional segment of trunk (body structure)	This cross‐sectional segment is bounded superiorly by a virtual horizontal plane at the level of the plane traversing the superior boundary of the iliac crest; and inferiorly it extends to the perineum and includes part but not necessarily the entire external genitalia. The segment includes the volume of the true and false pelvic cavities (and also part of the lower abdominal volume below the level of the virtual superior boundary). The volume includes the entire transverse thickness of the body over the longitudinal extent between the superior (upper) and inferior (lower) boundaries including the skin and subcutaneous tissue. The segment relates to the cross‐sectional or projectional volume as perceived by transmissive or emissive imaging.
	CLINICAL REGION	
818983003	Abdomen Structure of abdominopelvic cavity and/or content of abdominopelvic cavity and/or anterior abdominal wall (body structure)	This is considered the most commonly used clinical variant of ‘abdomen’ and relates to the space and content within the abdominopelvic cavity plus the anterior and lateral abdominal wall. The volume is bounded by, but excludes: superiorly the thoracic diaphragm; inferiorly the pelvic diaphragm and posteriorly the posterior wall of the abdomen proper: The pelvic component consists of the cavity of the true pelvis, which is bounded by, but excludes, the pelvic wall. Anteriorly this volume is bounded and includes the anterior abdominal (including the lateral abdominal wall).
818984009	Abdomen proper Structure of abdominopelvic cavity and/or intra‐abdominopelvic content and/or anterior abdominal wall excluding intra‐pelvic structure of true pelvis (body structure)	This is a clinical variant of ‘abdomen’ and relates to the ‘abdomen proper’ cavity which is defined as the abdominopelvic cavity and content, excluding the cavity and content of the true pelvis, plus the anterior and lateral abdominal wall (but excluding the posterior wall of the abdomen proper). This space and content is bounded by, but excludes: superiorly the thoracic diaphragm; inferiorly the superior pelvic aperture and posteriorly the posterior wall of abdomen proper. Anteriorly this volume is bounded and includes the anterior abdominal (including the lateral abdominal wall).
12921003	Pelvic region Structure of pelvis (body structure)	This structure, also termed the pelvic region, includes the wall, cavity and content of both the true and false pelvis; it consequently incorporates the entire bony pelvis; and inferiorly it is bounded and includes the pelvic diaphragm. The structure incorporates the complete pelvic wall; sacrococcygeal region (including the overlying skin and subcutaneous tissue); the contents of the false pelvic cavity; but in contrast to the ‘pelvic segment of trunk’ excludes the perineum, external genitalia.

## 4 CONCLUSIONS

The concepts presented document regional truncal volumes from an anatomical structural, segmental and cross‐sectional perspective. These different points of view have been integrated in a logical and comprehensive semantic model suitable for computer processing.

This approach aims to provide improved clarity when using ‘abdomen’ for indexing and searching for multi‐word description variants, with the single‐word ‘abdomen’ assigned to a single concept that supports most common clinical uses. The descriptions of the subtle variants are designed to assist users to choose the appropriate concept in anatomy, since anatomy is context neutral. For example, ‘CT of abdomen (procedure)’ is a valid and semantically clear description in the diagnostic imaging community because ‘abdomen’ means cross‐sectional in the context of computed tomography imaging, and explicitly excludes the cross‐sectional pelvis. It is unwarranted to have a description of ‘CT of cross‐sectional abdomen’, since a CT implies ‘cross‐sectional’, and it is also unnecessary to have a description of ‘CT of cross‐sectional abdomen without pelvis’, since it is understood in a CT imaging context that the cross‐sectional abdomen is disjoint from the cross‐sectional pelvis. By contrast, in a projectional radiography context, an ‘X‐Ray of abdomen (procedure)’ is understood to include the pelvis, as well as the walls and integument, and hence applies to the abdominopelvic cross‐sectional segment of trunk. Therefore, it is unnecessary to use these variant descriptions of abdomen when the context is clear.

The expression of constitutional parts of regional constructs within terminologies is challenging because the clinical mind‐set and imaging procedures generally begin from a regional perspective and focus down upon a constitutional part following examination and investigation. For example, the presentation of abdominal pain would prompt clinical examination of the abdomen (intra‐abdominopelvic structure and/or anterior abdominal wall); if examination reveals a posterior pulsating mass, this may be followed by a CT imaging procedure, which might confirm the presence of an abdominal aortic aneurysm on cross‐sectional slices through the abdomen.

Creating a formal semantic solution for reconciling pure anatomical and clinical regional perspectives has to be undertaken with caution, and evaluated with iterative impact validation, as partonomic representations can be fraught with difficulty. The law “that all and only concepts satisfying the definition of a higher‐level ‘ancestor’ concept are classified under it as descendants” has to be obeyed. If this law of integrity is violated, then reasoning will produce incorrect results (Rector et al., 2011).

One limitation of the approach described is that normal physiological changes in structures might affect their structural location and invalidate their semantic position within the terminology. For example, the gravid uterus gradually expands out of the true pelvic cavity into the false pelvic cavity and then encroaches on the cavity of the abdomen proper, potentially adversely affecting assumptions: Logic‐based reasoning might conclude that the uterus could not be responsible for any abnormality in the ‘abdomen proper cavity’ as the uterus normally resides in the true pelvis. In practice, this situation is largely mitigated by the fact that the clinical notion of the ‘Abdomen’ means the abdominopelvic cavity, which contains the uterus in both the native and ‘gravid state’.

It is hoped that the publication of this semantic model of clinical regions of the trunk will stimulate thought, as well as consideration of the varied use of the word ‘abdomen’ in different contexts. The model, by necessity, is detailed and explicit and its adoption although challenging should promote greater clarity in documentation, data entry, data retrieval, algorithm‐based decision support and artificial intelligence–based implementations.

## CONFLICT OF INTEREST

The authors declare that they have no competing interests.

## AUTHOR CONTRIBUTIONS

PB and YG conceived the study following DC’s critique of an earlier draft document. PB and YG wrote the first draft of the study and following review by DC further editing of the study was achieved collaboratively. PB was responsible for the creation and formatting of all the figures.
